# Consultant Pharmacist–Provider Collaboration in U.S. Assisted Living Facilities: A Pilot Study

**DOI:** 10.3390/pharmacy7010017

**Published:** 2019-02-01

**Authors:** Kenneth C. Hohmeier, Kelsey D. Frederick, Krishna Patel, Kristi Summers, Morgan Honeycutt

**Affiliations:** 1 College of Pharmacy, University of Tennessee, Nashville, TN 37211, USA; kdobbs1@uthsc.edu; 2 Macs Pharmacy, Knoxville, TN 37849, USA; kpatel37@uthsc.edu (K.P.); kristi@macspharmacy.com (K.S.); morgan@macspharmacy.com (M.H.)

**Keywords:** collaboration, assisted living facility, consultant pharmacist, diabetes, recommendations, medication therapy management

## Abstract

The purpose of this pilot study was to explore the impact of pharmacist-provided recommendations to general practitioners (GPs) of patients living in assisted living facilities (ALFs). A secondary objective of this study was to explore prescriber and ALF staff perceptions. This was a mixed-method, quasi experimental 1-group pre/post-test study with an explanatory qualitative arm using in-depth semi-structured interviews at five regional ALFs and one independent community pharmacy in East Tennessee. Residents older than 65 years of age, with confirmed diagnosis of Type II diabetes in the pharmacy’s medical record, taking anti-diabetic medication for at least 14 days and resident of affiliated ALF for at least past 30 days were enrolled. Phase 1 demonstrated a 35.1% (13/37 recommendations) acceptance rate of pharmacist recommendations. Phase 2 demonstrated a similar 31.3% acceptance rate of pharmacist recommendations (5/16 recommendations). The mean pre–post difference in average 30-day FBG was greater in the accepted group than the rejected recommendation group (−9.1 vs. −2.3 mg/dL). Pharmacist–GP collaboration in the ALF population was feasible and may improve the quality of patient care of these residents.

## 1. Introduction

Teamwork, communication, and collaboration among healthcare professionals is essential to implementing a team-based approach to providing safe and effective patient care. Benefits of collaboration include reduced medication errors and adverse events, improved patient health outcomes, increased communication with patients, reduced physician workload, and decreased costs [1,2]. Pharmacists in particular have unique training, expertise, and ability to make a lasting, positive impression on patient care through collaboration with physicians [1]. 

Evidence confirms the effectiveness of pharmacists in multidisciplinary teams, even if the collaborating pharmacist is external to the care-providing organization. A recent meta-analysis of 37 team-based intervention studies found significantly decreased blood pressure with pharmacist therapy recommendations or patient education [3,4]. Similarly, in the Collaboration Among Pharmacists and physicians To Improve Outcomes Now (CAPTION) study, physician–pharmacist collaboration was associated with a significant decrease in blood pressure compared to usual care [5]. 

With such growing evidence, national professional and governmental organizations are now calling for increased pharmacist–provider collaboration. The Centers for Disease Control and Prevention (CDC) supports physician–pharmacist collaboration in chronic disease state management to improve patient care and health outcomes [6]. A recent position statement released by the National Governors Association (NGA) also calls for increased pharmacist representation on patient care teams [7]. The Centers for Medicare and Medicaid Services (CMS) also recently recognized the valuable role of pharmacists in team-based care through their interpretation of the “incident to” rule, which allows a physician to bill for certain health care services that were provided by a pharmacist as long as those services are part of the existing patient care plan established by the physician and all requirements of the statute and regulations are met [8]. This confirms that pharmacists can bill for services incident to physician visits as part of the collaborative care team, encouraging such collaboration. 

Despite the growing call and evidence of benefit, current research demonstrates that implementing collaboration between pharmacists and physicians can be challenging. Some barriers include varied site locations, concern over loss of communication, perceived pharmacist competency, and reimbursement challenges [1,2,9]. Several facilitators in establishing efficient physician–pharmacist collaboration have been suggested, including proactively identifying patients who may benefit from pharmacist intervention; requiring appropriate training and credentialing of pharmacists; creating a set schedule for pharmacists to interview patients; provider awareness, experience, trust, and a clear role definition and guidelines; pharmacists’ access to patient records, and effective communication [1,9]. However, setting specific variables may influence particular barriers and facilitators to such collaboration and so current evidence may not be applicable across all collaborative relationships.

The increase in the prevalence of chronic disease that accompanies this aging population presents an opportunity and a need for healthcare teams to collaborate professionally [6,10,11]. Consultant pharmacists, pharmacists who focus on improving medication management in the long-term care (LTC) environment, provide expert knowledge on the use of medications or on the provision of pharmacy services to medical institutions, practices, and patients [12]. Consultant pharmacists now practice in a wide variety of settings including subacute care, psychiatric hospitals, hospice programs, in-home and community-based care, and assisted living facilities (ALFs). Similar to LTC, ALFs offer residents the ability to remain mostly independent, while providing close and modifiable access to nursing care, transportation, and supervised recreational activities. It is hypothesized that including consultant pharmacists on this patient care team may improve the lives of older adults by providing medication management, preventing adverse drug reactions, and resolving duplicative therapies [13]; however, there is lack of consistent state regulation supporting this role in the ALF setting [14,15]. Moreover, few studies have investigated the impact and feasibility of the consultant pharmacist–physician collaboration in this unique population. The present study aims to gather exploratory data on such impact and feasibility. 

The purpose of this pilot study was to explore the impact of pharmacist-provided recommendations to primary care providers (PCPs) of patients living in assisted living facilities (ALF). The secondary objective of this study was to explore prescriber and ALF staff perceptions of pharmacist recommendations for patients residing in ALF.

## 2. Materials and Methods 

### 2.1. Overview

This was a two-phase mixed-methods, pilot study exploring the novel role of the consultant pharmacist in the ALF setting. These two phases included an observational prospective study and a quasi-experimental 1-group pre/post-test study with an explanatory qualitative arm. It was set at 5 regional assisted living facilities located in eastern Tennessee. Patients within these ALFs had self-administered prescribed medications. The community pharmacy was an independent pharmacy offering traditional community pharmacy and LTC pharmacy services and was affiliated with a local College of Pharmacy for both graduate and post-graduate (i.e., residency) education. Pharmacists involved in the study were all post-graduate year 1 (PGY-1) community-based pharmacy residency trained. Additionally, a current resident was involved. ALF practitioners and staff had only a “traditional” relationship with study pharmacists, typical of most LTC pharmacies (e.g., phoning in prescription orders, asking drug questions, replying to pharmacist questions). No training was provided to ALF practitioners or staff prior to the study’s launch. 

The purpose of phase 1 was to determine the feasibility and acceptability of proactive pharmacist-delivered recommendations made to general practitioners (GPs) in ALFs in Knoxville, TN. In the initial phase, a consultant pharmacist provided recommendations to the corresponding GP for patients with uncontrolled diabetes based on fasting blood glucose levels (FPG). Acceptance, rejection, and recommendations without a response were tracked. Based on preliminary data from phase 1, phase 2 was launched to measure the impact of proactive pharmacist-delivered recommendations on fasting blood glucose (FBG) for those patients with uncontrolled diabetes based on FBG. Throughout both phases, in-depth semi-structured interviews captured thoughts, feelings, and perceptions of the pharmacists’ role in the ALF setting and related pharmacist-delivered recommendation making. Phase 1 took place from January 2016 to June 2017. Phase 2 took place from January 2017 to June 2018. This study was approved by the institutional review board at the University of Tennessee Health Science Center (UTHSC).

### 2.2. Phase 1 

Utilizing QuickMar electronic health record (EHR) software (Eagle, ID), patient reports were created from which to enroll ALF residents. Inclusion criteria were older than 65 years of age, diagnosis of diabetes within the pharmacy’s medical records, residence in the ALF for at least 30 days, and current use of an antidiabetic medication for at least 14 days. Patients were excluded if they were transitioned into an LTC facility. Upon enrollment, blood glucose readings for the previous 30 days were collected for each resident. Fasting and postprandial blood glucose target ranges were based on the most current American Diabetes Association (ADA) guidelines (Table 1) [16]. Upon review of the patient data, pharmacist-initiated recommendations were documented and faxed to the patient’s corresponding GPs. Intervention fax forms specifically detailed dates of the resident’s uncontrolled readings and the proposed recommendation.

### 2.3. Phase 2

For phase 2, the EHR was again used to identify patients from which to enroll ALF residents. Inclusion and exclusion criteria matched phase 1. Upon ALF resident enrollment, fasting blood glucose (FBG) readings for the previous 30 days were evaluated for each resident according to ADA guidelines [16]). Based on these guidelines and professional judgement, pharmacists then faxed corresponding ALFs and prescribers of the residents with recommendations. After the recommendations were accepted or denied by the prescribers, the pharmacist obtained fasting blood glucose for 30 days post-recommendation. 

Quantitative data was analyzed using SPSS 23 (Armonk, NY, USA). Descriptive statistics are reported. For phase 2, only those recommendations which were accepted or rejected were included in analysis, as these were the only recommendations known to have been reviewed by the GP. 

### 2.4. Explanatory Qualitative Follow-Up

To determine the prescribers’ thoughts on pharmacist recommendations, a sequential, explanatory qualitative arm followed quantitative data collection using semi-structured, in-depth interviews. Respondents were recruited from a convenience sample of GPs and staff at the ALF facilities (e.g., physicians, nurse practitioners, nurses) involved in the recommendation process. 

To collect ALF practitioner and staff perceptions, an expert panel of site pharmacists, University researchers, and a pharmacy resident developed an interview guide consisting of 5 domains. The 5 domains included:

- Pharmacists’ current role in ALF
- Provider’s current role in ALF
- Future of healthcare related to ALF
- Diabetic medication recommendations
- Pearls: advice to other pharmacists leading similar programs

The interviews were audio recorded then transcribed. Coding was performed deductively using the Conceptual Model of GP–pharmacist Collaboration [17]. This conceptual model was developed from previous and well-established conceptual models of the stages and characteristics of collaboration between different health and/or social care professions, and adapted specifically to the community pharmacy setting. To date, this is the only collaborative care conceptual model which specifically applies established collaboration theory to the community pharmacy. Three sequential stages of collaboration are detailed and comprise (1) Isolation, (2) Communication, and (3) Collaboration. Seven factors and their relation to each stage are also described in the model, and comprise locality, service provision, trust, knowing each other, communication, professional roles, and professional respect. Two coders with a background in qualitative research performed the analysis by hand. Interviews continued concurrently with data analysis until a point of saturation was achieved, at which point no new information emerged from interviews. 

## 3. Results

### 3.1. Phase 1

Within the five assisted living facilities selected, 41 residents were identified with a diabetes diagnosis. Of those 41 residents, 39 qualified for enrollment in the study. Of these, a total of 37 recommendations were made for these residents. 

Of the 37 interventions made for the ALF residents, 13 recommendations (35.1%) were accepted by prescribers. Six recommendations (16.2%) were denied, and the remaining 18 recommendations (48.7%) resulted in no response from GPs. 

### 3.2. Phase 2

Within the four ALF facilities selected, 22 residents were enrolled and 16 patients had corresponding pharmacist-initiated recommendations for therapy change. In total, 5 recommendations were accepted (31.3%), 4 were denied (25%), and 7 resulted in no response from GPs (43.8%). Demographic data can be found in Table 2. The mean pre–post difference in average 30-day FBG was greater in the accepted group than the rejected recommendation group (−9.1 vs. −2.3 mg/dL). 

### 3.3. Explanatory Qualitative Follow-Up

In total, there were five respondents interviewed. Interviews took place between January 2016 and June 2018. Respondent titles included physicians, nurse practitioners, administrators, and nurses. There were two overarching themes on pharmacist–GP collaboration within the ALF setting: Stages of Collaboration for Pharmacists and GPs in ALFs and Factors Influencing Collaboration for Pharmacists and GPs in ALFs 

#### 3.3.1. Stages of Collaboration for Pharmacists and GPs in ALFs

Cross-case analysis found that collaboration level was not consistent across institutions or GPs. In general, lower levels of collaboration were noted when GPs were unfamiliar with the pharmacist (Level 1—Isolation Stage and Level 2—Communication Stage). Conversely, in those interviews which noted a higher degree of collaboration, the GP or GP’s staff identified the pharmacist by name typically, emphasizing the importance of an established relationship between the two (Level 3—Collaboration Phase). Several factors influenced this familiarity and subsequently higher level of collaboration.

#### 3.3.2. Factors Influencing Collaboration for Pharmacists and GPs in ALFs

Stages of collaboration were impacted by factors ascribed to the Conceptual Model of Pharmacist–GP Collaboration [17]. Most professional roles revolved around ensuring appropriate medication use for those medications already being prescribed by GPs within the ALFs. Respondents also felt that pharmacists could provide assistance in answering medication-related questions.

“For our facility, they’re very involved. We’re on the phone with them, our nurses are on the phone with them a lot, with questions. I find it extremely helpful.”

(Administrator 1)

For those respondents who reported a “high work load,” the pharmacist’s role included helping to reduce stress load. Although GPs felt some workload resolution through collaboration with the pharmacist, this was not noted on the administrative personnel and nursing side.

“Well, [workload’s] a little high. But primary care is really hard, because we address so many issues. I think I came in on the back-end of [pharmacist-provided recommendations], and I might have gotten maybe one or two things from her. But, I thought it was wonderful. Like, right now I would like to have [the pharmacist review] several of [my patients].”

(GP 2)

“The workload is manageable. I don’t think [pharmacist-provided recommendations] would make a difference.”

(Administrator 1)

The most common specific services which the respondents associated the pharmacist with included answering questions, making recommendations, and troubleshooting insurance issues. Nurses and wellness administrators struggled to identify the value of the pharmacist outside of traditional roles, and generally were not aware of currently existing consultant pharmacist roles. Future roles for the pharmacist in ALFs included working with transitions of care and providing diabetes self-management education.

“You know in an ideal world, to me, for consulting pharmacist, I would like for them to at least every six months to be able to review all the residents in the facility, to see what medications they’re on, any recommendations they have.”

(GP 1)

Respondents noted that trust and “knowing each other” was important to facilitating recommendation approval. Specifically, the development of a rapport between the GP and pharmacist was described as a facilitator.

“…[recommendation approval] just depends with each doctor, and as you work with them and you get a rapport with them [sic] things get done.”

(Nurse 1)

Another key facilitator was communication, and respondents valued pharmacists who were easy to access and who were available “on call.” Telephone consultations with straightforward, quick answers and recommendations were preferred. Beyond this, respondents noted the need for consistency of this level of communication, regardless of the day of the week or hour of the day.

“Like I said, we have always had a great relationship. Anything I need, I just take up a phone, call [pharmacist name 1], or [pharmacist name 2] to get something done. [sic] We just have a good relationship and good rapport with everybody. If we need anything we know how to get it.”

(GP 2)

## 4. Discussion

Multidisciplinary collaboration is key to improving patient outcomes due to the complexity of ALF patients’ mediation therapies and chronic conditions [12]. This study suggests that pharmacists can improve diabetes outcomes for patients in ALFs through personalized, timely medication management in a feasible team-based model of care. Pharmacists are among the most accessible of health care professionals and are well equipped to make a positive, lasting impact on patient care and quality of life through communication and coordination with health care team members [6]. Similar acceptance rates were exhibited between phases of this study. Phase 1 determined the acceptability of a consultant pharmacist’s recommendations made to GPs regarding patients’ blood glucose control, which turned out to be an acceptance rate of approximately 35%. Phase 2 observed the impact of these recommendations on FBG for patients with uncontrolled diabetes, resulting in an acceptance rate of approximately 31%. This acceptance rate is similar to other published studies in LTC settings [18,19], but lower than in other settings [20,21].

Other studies exhibited similar effectiveness of collaboration between pharmacists and physicians. For example, a reduction in the number of potentially inappropriate medication orders (based on Beers’ criteria) for elderly patients residing in ALFs through consultant pharmacist medication review and recommendations has been previously reported [22]. Another study found improved chronic medication management through physician–pharmacist collaboration with a 47% acceptance rate of pharmacist therapy recommendations in the community setting [23]. Although the present study also reports surrogate endpoints to improved patient outcomes within the ALF setting, when taken together with similar studies one begins to see the positive association between pharmacist–GP collaboration. This trend warrants further investigation both in terms of major endpoints such as morbidity and mortality, as well as qualitative endpoints such as successful facilitators of implementing such collaborative practice.

Strengthening the partnership between physicians and pharmacists benefits patients, as well as both professions. Perceived benefits include improving means of communication, reduced medication-related problems, reduced physician workload, improved patient outcomes and quality of care, and decreased unnecessary treatment and medication waste [1,2]. Additionally, pharmacists’ specific drug knowledge and training complements the skills and knowledge of physicians, facilitating improved patient care and quality of life [6,24]. As was seen in our study, benefits within the ALF facility included meeting guideline-recommended standards of care for patients with diabetes.

The Canadian Interprofessional Health Collaborative (CIHC) suggests six essential competencies for successful interprofessional collaboration: interprofessional communication, patient/client/family/community-centered care, role clarification, team functioning, collaborative leadership, and interprofessional conflict resolution [25]. These skills are developed over time as the collaborative relationship builds. There are several similarities between CIHC’s model and our qualitative results, suggesting that collaboration between consultant pharmacists and ALF providers is not dissimilar from other healthcare settings.

While examples of physician–pharmacist collaboration models do currently exist, a significant opportunity to expand pharmacist involvement in team-based care still remains, particularly in the ALF setting. More ALFs should support the development of physician–pharmacist collaborative care to help patients better manage their medications and overall health (23). Future research is needed to further explore the pharmacist’s role in this setting. 

Due to the pilot study design, there were several limitations. This study is non-randomized, which yields non-equivalent test groups and limits the generalizability of the results. This, in turn, reduces internal validity and may render statistical analyses meaningless. Furthermore, the information is considered pilot data, and additional data is needed. This study is quasi-experimental, meaning its variables are less controlled and its conclusions about causality are less definitive by nature [26]. Finally, this study occurred at a single community pharmacy in eastern Tennessee with a small number. To offset some of these threats to validity, a mixed-methods approach and qualitative data did support findings from quantitative results. 

## 5. Conclusions

Pharmacist–GP collaboration in the ALF population was shown to be feasible and may improve the quality of patient care of these residents. Future studies should expand on these initial findings to identify the pharmacist’s role in this novel patient care setting.

## Figures and Tables

**Table 1:** American Diabetes Association (ADA): Treatment goals for diabetes.

Patent Characteristics/Health Status)	Fasting or Pre-Prandial Glucose (mg/dL)	Bedtime Glucose (mg/dL)
Healthy (Few coexisting chronic illnesses, intact cognitive and functional status)	90–130	90–150
Complex/intermediate (Multiple coexisting chronic illnesses or 2+ instrumental ADL impairments or mild to moderate cognitive impairment)	90–150	100–180
Very complex/poor health (Long-term care or end-stage chronic illnesses or moderate to severe cognitive)	100–180	110–200

**Table 2:** Phase 2 patient demographic data by recommendation result.

Demographics		
	Accepted Recommendations (n = 5)	Denied Recommendations (n = 4)
Age (>65)	82–91	82–91
Sex	Female 80% (n = 4)	Female 75% (n = 3)
Number of diabetes medications	Avg. = 2.2	Avg. = 2
